# Difference in Personality Traits and Symptom Intensity According to the Trigger-Based Classification of Throwing Yips in Baseball Players

**DOI:** 10.3389/fspor.2021.652792

**Published:** 2021-08-25

**Authors:** Toshiyuki Aoyama, Kazumichi Ae, Hiroto Souma, Kazuhiro Miyata, Kazuhiro Kajita, Takashi Kawamura, Koichi Iwai

**Affiliations:** ^1^Department of Physical Therapy, Ibaraki Prefectural University of Health Sciences, Ami-machi, Japan; ^2^Department of Sports Rehabilitation, Gakusai Hospital, Nakagyo-ku, Japan; ^3^Faculty of Health and Sport Sciences, University of Tsukuba, Tukuba, Japan; ^4^Educational Development Center, Kyoto University of Advanced Science, Kameoka, Japan; ^5^Center for Humanities and Sciences, Ibaraki Prefectural University of Health Sciences, Ami-machi, Japan

**Keywords:** baseball yips, big five personality traits, neuroticism, agreeableness, classification

## Abstract

The triggers of initial onset of yips symptoms can be broadly divided into psychological and non-psychological factors; however, a trigger-based classification of yips has not been established. This study aims to obtain insight into the prevention of yips by clarifying whether there are differences in symptoms and personality traits according to a trigger-based classification of yips in baseball players. A total of 107 college baseball players responded to a questionnaire assessing the presence or absence of yips and its symptoms. They were classified into the psychologically triggered yips group, the non-psychologically triggered yips group, and the non-yips group based on the presence or absence of yips and the triggers of its initial onset. Additionally, we compared whether personality traits examined by the NEO Five-Factor Inventory differed across these three groups. The psychologically triggered yips group had significantly higher agreeableness scores compared with the non-yips group, whereas the non-psychologically triggered yips group had significantly higher neuroticism scores compared with the psychologically triggered yips group. In the non-psychologically triggered yips group, there was a significantly higher frequency of throwing errors than in the psychologically triggered yips group, with a tendency to develop yips symptoms gradually. Since the trigger-based classification of yips is closely related to the strength of the yips symptoms and the players' personality traits, the results of this study contribute to a better understanding of the symptoms of yips and establishment of the prevention of yips. Large prospective studies are necessary to determine the causal relationship between a trigger-based classification of yips and the personality traits and symptoms of athletes with yips.

## Introduction

The “yips” phenomenon is a psycho-neuromuscular disorder characterized by involuntary movements that disrupt the execution of automatic fine motor behavior (Smith et al., 2000; Bawden and Maynard, 2001; Clarke et al., 2015). Yips have been reported to occur in athletes of various sports including golf (McDaniel et al., 1989; Smith et al., 2000; Adler et al., 2005, 2011, 2018; Stinear et al., 2006; Dhungana and Jankovic, 2013; Klämpfl et al., 2013a,b; Ioannou et al., 2018), cricket (Bawden and Maynard, 2001; Roberts et al., 2013), archery (Clarke et al., 2019), baseball (Jones et al., 2017; Smith et al., 2017; Nakane et al., 2018; Aoyama et al., 2021), and darts (Roberts et al., 2013). According to a systematic review by Clarke et al. (2015), 28–54% of low handicap golfers have experienced yips. Smith et al. (2003) proposed classifying yips-affected players into two categories based on their symptoms: Players with physical symptoms such as “jerking” or “freezing” were classified as focal dystonia (type I), whereas players with psychological symptoms such as “anxiety” were classified as choking (Type II). This symptom-based classification is very beneficial when choosing a treatment for yips. For example, in Type I yips, botulinum toxin injection and oral medication are recommended, whereas psychological intervention is proposed as a treatment for Type II yips (Stinear et al., 2006; Adler et al., 2011; Ioannou et al., 2018). Conversely, when aiming to prevent yips, the causes, i.e., the episodes that trigger initial onset of yips, must be evaluated in addition to the symptoms themselves. A previous study indicated that 75% of yips-affected golfers began to exhibit the symptoms during a tournament (McDaniel et al., 1989). Therefore, psychological factors such as extreme pressure or anxiety might strongly influence the initial onset of yips. However, psychological factors are not the only triggers of yips. McDaniel et al. (1989) reported that non-psychological factors such as workload may be involved in the development of yips. Additionally, in focal dystonia patients, non-psychological factors such as workload, changes in technique, and prolonged pain have been reported to be involved in symptom onset (Altenmüller and Jabusch, 2009, 2010). As described above, yips triggers can be broadly divided into psychological and non-psychological factors, but yips have not yet been classified according to their triggers. This type of classification could help develop preventive methods for each type of yips trigger. Therefore, the primary purpose of this study was to establish a classification of yips based on the episode that triggered the first appearance of yips symptoms.

Previous studies have shown that task-specific dystonia, which is a subtype of yips, is related to individual personality. Enders et al. (2011) investigated the big five personality traits in focal dystonia patients using the NEO Five-Factor Inventory (NEO-FFI), which consists of 60 items (Costa and McCare, 1992). They revealed that patients with musician's dystonia have significantly higher neuroticism scores, which is an indicator of emotional instability and includes a tendency to experience negative emotions and vulnerability to stress, than non-musicians or healthy musicians. Therefore, personality traits might be related to yips, which is thought to have a common pathophysiology with task-specific dystonia. Only one study has investigated the relationship between yips and the big five personality traits (Clarke et al., 2019). They did not identify a significant difference in neuroticism scores determined with the Big Five Inventory-10 (BFI-10) between a yips group and non-yips group. However, the BFI-10 only includes two items for each factor. Therefore, although the BFI-10 is suitable for quick and easy investigations (Rammstedt and John, 2007), its reliability is somewhat questionable (Clarke et al., 2019). Furthermore, the relationship between trigger-based classification of yips and big five personality traits has not been clarified. In this context, the secondary purpose of the current study was to investigate whether the big five personality traits differed according to trigger-based classification of yips using NEO-FFI, which is a reliable index consisting of 12 items per dimension (Costa and McCare, 1992). We hypothesized that the personality traits of athletes classified by their triggers of yips would differ.

Symptoms of yips in golfers are more pronounced during short putts of ~1–4 feet (Smith et al., 2000). In our preliminary report (Aoyama et al., 2021), which investigated throwing yips in baseball players, symptoms appeared to be stronger when throwing at a short distance of ≤ 20 m. Such task-specific symptoms are found not only in athletes with yips but also in patients with focal dystonia (Altenmüller and Jabusch, 2009, 2010). Furthermore, symptoms of yips fluctuate with psychological state. McDaniel et al. (1989) reported that yips symptoms increase in high-pressure situations, such as tournaments, compared to low-pressure situations such as practice. However, it is unclear whether such context-dependent changes in the symptom intensity of yips, i.e., changes in symptoms due to throwing distance and psychological pressure, differ depending on the triggers of the yips. Therefore, the tertiary purpose of this study was to clarify whether there are differences in the effects of throwing distance and psychological pressure on throwing performance according to trigger-based classification of yips.

The overall aim of the present study was to obtain insights into the mechanisms and prevention of yips by clarifying personality traits and characteristics of the symptoms based on the new classification of yips in baseball athletes.

## Methods

### Participants

A total of 107 college baseball players from the First Division of the Tokyo Metropolitan Area Collegiate Baseball League participated in this study. The participants had an average (SD) age of 20.3 (1.3) years and an average of 12.5 (2.1) years of baseball experience. Based on the effect size obtained from a previous study using the NEO-FFI, the sample size was estimated by performing a power analysis using G*Power with a power of 0.8 and an alpha of 0.05 (Clarke et al., 2019). This study was performed in accordance with the recommendations of the Declaration of Helsinki established by the World Medical Association. The protocol was approved by the local ethics committee of the Ibaraki Prefectural University of Health Sciences (Approved number: 876), and informed consent for participation in this study was obtained from all participants.

### Questionnaires

An anonymous self-administered questionnaire was distributed to the participants in August 2019. After completing this questionnaire, the participants submitted it to a researcher who was not affiliated with the players. The questionnaire content was the same as that used in our previous study (Aoyama et al., 2021). The questionnaire was originally developed based on previous reports in yips-affected golfers (McDaniel et al., 1989; Smith et al., 2000) and consultation with the authors, including coaches and a physical therapist who have experience dealing with yips-affected baseball players, a neuroscience researcher (physical therapist) who has physical therapy experience in focal dystonia, and a sports biomechanics researcher who specializes in baseball. The survey data included age, baseball experience, throwing arm, and the presence or absence of yips symptoms; these data were collected from all participants. We classified the players as having yips if they answered “Yes” to a question about the subjective experience of the yips symptoms (Have you ever suddenly or gradually been unable to control the ball as you would like and continued to make throwing errors that your partner would not be able to catch?) and whose symptoms persisted for at least 1 month (Aoyama et al., 2021). We excluded three participants from this study who had a subjective experience of yips symptoms that lasted <1 month. Players who had experienced yips symptoms answered whether there was an episode that triggered initial onset of symptoms. If there was a triggering episode, they selected the most applicable trigger from the following candidates: “pain,” “experience of throwing errors,” “anxiety about throwing,” “reprimand from others,” and “change in throwing mechanics.” If none of these opinions were applicable, the players described a specific trigger. With respect to symptom progression, the players answered whether the discreet symptoms became gradually more intense (gradual onset) or whether clear symptoms suddenly occurred (acute onset). Additionally, the duration of yips, age at initial onset, and whether symptoms were still present were collected. We also investigated whether the severity of yips symptoms, i.e., the frequency of throwing errors, varied by throwing distance and trigger-based classification of yips. The throwing distance was classified into five categories: <10, 10–20, 20 m to between base distance, between-base distance to 50, and >50 m. Players who had experienced yips selected the frequency of throwing errors at each throwing distance at the time when their symptoms were worst based on the following five-point ordinal scale: 0 = never (no symptoms), 1 = seldom (1–2 out of 10 throws), 3 = occasionally (3–5 out of 10 throws), 4 = frequently (6–8 out of 10 throws), and 5 = almost always (≥9 out of 10 throws).

The big five personality traits were assessed with the NEO-FFI. The following five dimensions were assessed: interpersonal interactions (extraversion); the tendency to seek out new experiences (openness); emotional instability, e.g., anxiety, depression, and self-consciousness (neuroticism); goal-directed behavior and organizations (conscientiousness); and social harmony and concern for cooperation (agreeableness) (Costa and McCare, 1992). The NEO-FFI consists of 12 items per dimension, for a total of 60 items.

### Analysis

We divided the participants into four groups based on their episodes that triggered the initial onset of yips symptoms: psychologically triggered yips group, non-psychologically triggered yips group, pain-triggered yips group, and non-yips group. Players were classified into psychologically triggered yips groups when episodes strongly associated with psychological factors such as “experience of throwing error,” “anxiety about throwing,” and “reprimands from others” triggered initial onset of yips. Players who were triggered by a change in throwing mechanics and who had no specific triggers for initial onset of yips, i.e., psychological factors did not trigger the initial onset of yips symptoms, were classified into the non-psychologically triggered yips group. Players who developed yips due to pain were not included in the statistical analysis because they could not be classified into either the psychological or non-psychological yips groups, and their numbers were small. All statistical analyses were performed using SPSS version 26 statistical software (IBM, Armonk, NY, USA). The level of statistical significance was *p* < 0.05. We performed the Shapiro-Wilk test to examine the null hypothesis stating that the obtained data were normally distributed. Furthermore, Leven's tests were performed to examine the equality of variance. When assumptions of normality or equality of variances were not met, non-parametric tests were used for the statistical analysis. We used generalized linear mixed models (GLMM) to examine the effect of trigger-based classification of yips and throwing distance on the frequency of throwing errors, assuming a multinomial distribution of the dependent variable with a cumulative logit link function. Fixed effects included classification of yips and throwing distance, and the random effects included participants in the model. Sequential Bonferroni correction was used in *post hoc* comparisons. A Fisher-Freeman-Halton test was conducted to examine whether worsening of symptoms in psychologically stressful situations, such as games, differed between two yips groups. The five subscores of NEO-FFI were analyzed using multivariate analysis of variance (MANOVA) with one intersubject factor “group” (psychologically triggered yips group, non-psychologically triggered yip group, and non-yips group). If an overall significant effect was obtained by MANOVA, a separate ANOVA was conducted. Tukey's *post hoc* tests were used for multiple comparisons. The effect size (partial η2) was also calculated.

## Results

Participants were classified into the psychologically triggered yips group (*n* = 19), the non-psychologically triggered yips group (*n* = 25), the pain-triggered yips group (*n* = 5), and the non-yips group (*n* = 55) based on the trigger of the initial onset of yips. The triggers of the psychologically triggered yips group included “experience of throwing error” (*n* = 11), “anxiety about throwing” (*n* = 5), “reprimand from others” (*n* = 2), and “fear-related throwing experience (the experience of being hit by a batted ball after throwing)” (*n* = 1). The non-psychologically triggered yips group consisted of 3 players triggered by a “change in throwing mechanics” and 22 players with no specific triggers.

The participants' characteristics are shown in Table 1. There were no significant differences in age [*H*(2) = 1.193, *p* = 0.551] and baseball experience [*H*(2) = 0.106, *p* = 0.948] among the psychologically triggered yips, non-psychologically triggered yips, and non-yips groups. The Fisher-Freeman-Halton test revealed no statistically significant difference in throwing arm among these three groups (*p* = 0.776). The Mann-Whitney U test showed that there were no significant differences in age at initial onset (*z* = 0.808, *p* = 0.419) and duration of yips (*z* = 0.444, *p* = 0.657) between the psychologically triggered yips and non-psychologically triggered yips groups. There was no significant difference between the two yips groups regarding whether yips symptoms were still present [χ^2^_(1)_ = 2.087, *p* = 0.149]. In the psychologically triggered yips group, nine players displayed acute onset of symptoms while 10 players displayed a gradual onset of symptoms. Conversely, in the non-psychologically triggered yips group, 80% (20 out of 25 players) displayed a gradual onset. Although the p value did not reach statistical significance, the Chi-square test showed that the development of symptoms tended to differ between the psychologically triggered yips group and the non-psychologically triggered yips group [$\chi_{\left(1\right)}^{2}$ = 3.727, *p* = 0.054].

**Table 1:** Participant characteristics.

	**Psychologically triggered yips group**	**Non-psychologically triggered yips group**	**Non-yips group**	***p***
*n*	19	25	55	
Age	21 (20–21)	20 (20–21)	20 (19–21)	0.551
Baseball experience (Year)	13 (11–14)	12 (11–14)	12 (11–14)	0.948
Throwing arm (R/L)	17/2	23–2	47/8	0.776
Initial onset (Age)	17 (15–18.5)	17 (16–19)	–	0.419
Duration of yips (Month)	10 (1.5–37)	5.5 (2.25–11.5)	–	0.657
Symptoms at present (+/–)	11/8	9/16	–	0.149
Development of symptoms (Acute/Gradual)	9/10	5/20	–	0.054

*The Kruskal-Wallis tests were used for Age and Baseball experience: Median (first quartile-third quartile)*.

*Fisher-Freeman-Halton test was used for Throwing arm: n*.

*The Mann-Whitney U test were used for Initial onset and Duration of yips: Median (first quartile-third quartile)*.

*The chi-square tests were used for Symptoms at present and Development of symptoms: n*.

The relationship between the frequency of throwing errors and throwing distance in both yips groups is shown in Table 2. The GLMM showed that corrected model was statistically significant [*F*_(3, 207)_ = 7.294, *p* < 0.001], and the interaction between the yips classification and throwing distance was not significant [*F*_(4, 207)_ = 0.595, *p* = 0.667] (Table 3). There was a significant fixed effect for the yips classification [*F*_(1, 207)_ = 5.591, *p* = 0.019]. The frequency of throwing error was significantly higher in the non-psychologically triggered yips group than in the psychologically triggered yips group. There was a significant fixed effect for the throwing distance [*F*_(4, 207)_ = 14.734, *p* < 0.001]. In addition, the multiple comparison test revealed that frequency of the throwing errors was significantly higher in short-distance throwing (<10 m, from 10 to 20 m and from 20 m to between base distance) compared with long distance (>50 m) throwing (*t* = 5.047, *p* < 0.001, *t* = 5.217, *p* < 0.001, *t* = 2.855, *p* = 0.005, respectively).

**Table 2:** Relationship between frequency of throwing errors and throwing distance.

	**Psychologically triggered yips group**	**Non-psychologically triggered yips group**
Under 10 m: Median (first–third quartile)	1 (0–2)	2 (1–3)
0 [Never: *n* (%)]	8 (42.1)	4 (16.0)
1 [Seldom: *n* (%)]	4 (21.1)	6 (24.0)
2 [Occasionally: *n* (%)]	3 (15.8)	8 (32.0)
3 [Frequently: *n* (%)]	4 (21.1)	5 (20.0)
4 [Almost always: *n* (%)]	0 (0)	2 (8.0)
From 10 to 20 m: Median (first–third quartile)	1 (0–2)	2 (1–3)
0 [Never: *n* (%)]	8 (42.1)	5 (20.0)
1 [Seldom: *n* (%)]	4 (21.1)	5 (20.0)
2 [Occasionally: *n* (%)]	3 (15.8)	6 (24.0)
3 [Frequently: *n* (%)]	3 (15.8)	7 (28.0)
4 [Almost always: *n* (%)]	1 (5.3)	2 (8.0)
From 20 m to between base distance: Median (first–third quartile)	0 (0–1)	1 (0–2.5)
0 [Never: *n* (%)]	12 (63.2)	11 (44.0)
1 [Seldom: *n* (%)]	3 (15.8)	5 (20.0)
2 [Occasionally: *n* (%)]	3 (15.8)	3 (12.0)
3 [Frequently: *n* (%)]	1 (5.3)	4 (16.0)
4 [Almost always: *n* (%)]	0 (0)	2 (8.0)
From between base distance to 50 m: Median (first–third quartile)	0 (0–1)	1 (0–2)
0 [Never: *n* (%)]	14 (73.7)	11 (44.0)
1 [Seldom: *n* (%)]	4 (21.1)	7 (28.0)
2 [Occasionally: *n* (%)]	1 (5.3)	5 (20.0)
3 [Frequently: *n* (%)]	0 (0)	1 (4.0)
4 [Almost always: *n* (%)]	0 (0)	1 (4.0)
Over 50 m: Median (first–third quartile)	0 (0–0)	0 (0–1)
0 [Never: *n* (%)]	18 (94.7)	15 (60.0)
1 [Seldom: *n* (%)]	0 (0)	7 (28.0)
2 [Occasionally: *n* (%)]	0 (0)	1 (4.0)
3 [Frequently: *n* (%)]	1 (5.3)	1 (4.0)
4 [Almost always: *n* (%)]	0 (0)	1 (4.0)

**Table 3:** Statistical results of GLMM for the effect of yips classification and throwing distance on the frequency of the throwing errors.

**Fixed effect**	***F*-value**	**Degree of freedom**	***p***
Yips classification	7.294	1, 207	0.019^*^
Throwing distance	14.737	4, 207	<0.001^*^
Yips classification × Throwing distance	0.595	4, 207	0.667

*The significant difference of the fixed effect was indicated with an asterisk (p < 0.05)*.

There was no significant difference between the two yips groups in the deterioration of throwing errors under stressful conditions such as games (*p* = 0.463, Table 4).

**Table 4:** Response to a question assessing whether the yips symptoms are more intense in games than in practice.

	**Psychologically triggered yips group**	**Non-psychologically triggered yips group**	***p***
Median (first–third quartile)	1 (0–2)	1 (0–2)	
0 [Disagree: *n* (%)]	7 (42.1)	6 (24.0)	
1 [Slightly agree: *n* (%)]	4 (15.8)	8 (32.0)	0.463
2 [Agree: *n* (%)]	5 (26.3)	4 (16.0)	
3 [Strongly agree: *n* (%)]	3 (15.8)	5 (20.0)	

*p value indicating the results of Fisher-Freeman-Halton test*.

The results of MANOVA (Table 5) revealed that there was a significant difference in the subscores of NEO-FFI among three groups (Pillai's trace = 0.193, *p* = 0.037). The separate ANOVA indicated that the agreeableness score and neuroticism score differed significantly among the three groups [agreeableness: *F*_(2, 96)_ = 4.305, *p* = 0.016; neuroticism: *F*_(2, 96)_ = 3.420, *p* = 0.037]. The *post hoc* test showed that the agreeableness score was significantly higher in the psychologically triggered yips group compared with the non-yips group. Conversely, the neuroticism score was significantly higher in the non-psychologically triggered yips group compared with the psychologically triggered yips group. The other three dimensions did not differ significantly among the three groups [extraversion: *F*_(2, 96)_ = 0.081, *p* = 0.922; openness: *F*_(2, 96)_ = 0.872, *p* = 0.422; conscientiousness: *F*_(2, 96)_ = 2.225, *p* = 0.114].

**Table 5:** Results of the NEO-FFI.

	**a. Psychologically triggered yips group**	**b. Non-psychologically triggered yips group**	**c. Non-yips group**	***p***	***ηp^**2**^***
Neuroticism	22.5 (6.9)*^b^	27.9 (6.9)*^a^	25.3 (6.7)	0.037*	0.067
Extraversion	28.5 (6.6)	28.4 (5.5)	28.0 (6.9)	0.922	0.002
Openness	27.6 (4.0)	29.2 (5.3)	27.3 (6.9)	0.422	0.018
Agreeableness	33.2 (6.4)*^c^	30.1 (4.5)	28.6 (6.3)*^a^	0.016*	0.082
Conscientiousness	30.2 (6.0)	26.9 (4.6)	28.9 (5.4)	0.114	0.044

*Mean (SD) score of each NEO-FFI sub-scale. The significant differences in the post hoc analysis are indicated with an asterisk (p < 0.05) and different letters (a; vs. Psychologically triggered yips group, b; vs. Non-psychologically triggered yips group, c; vs. Non-yips group)*.

## Discussion

The present study focused on triggers of the initial onset of throwing yips in baseball players and divided the triggers into psychological and non-psychological factors. The most striking finding of the present study was the difference in personality traits between the psychologically and non-psychologically triggered yips groups; i.e., the former had significantly higher agreeableness scores compared with the non-yips group, while the latter showed significantly higher neuroticism scores compared with the psychologically triggered yips group. Furthermore, the non-psychologically triggered yips group showed a significantly higher frequency of throwing errors and a tendency toward gradual development of yips symptoms compared with the psychologically-triggered yips group. Thus, the trigger-based classification of yips could potentially contribute to the prevention of yips because it is closely related to the symptoms and personality traits of the players.

In 19 out of 49 (38.8%) yips-affected baseball players, psychological factors such as throwing errors, anxiety about throwing, and reprimand from others led to initial onset of yips. Conversely, 25 out of 49 (51.0%) yips-affected athletes developed yips due to non-psychological factors, including those who had no specific trigger for initial onset and those who developed yips after a change in throwing mechanics. A previous study reported that 75% of yips-affected golfers began to develop symptoms in a tournament in which psychological pressure was assumed to be high, suggesting that psychological factors account for a large proportion of the triggers of golfers' yips (McDaniel et al., 1989). Therefore, involvement of psychological factors in the initial onset of throwing yips in baseball is likely to be lower than that of golfers' yips. Although the reason for this difference is unclear from the current study, it may be a result of the different characteristics of the sports, such as required skills (i.e., open skills or closed skills) and regulations (individual or team sports). Regardless, since the initial onset of yips in each sport may differ depending on characteristics of the sport, it is important to investigate them to prevent yips.

10% of the yips-affected baseball players developed yips because of pain. Jankovic et al. reported that some patients develop focal dystonia triggered by pain (Jankovic and Van Der Linden, 1988). Therefore, although infrequent, pain may be one of the triggers for the initial onset of both yips and focal dystonia. Throwing yips that occur after pain can be triggered by both psychological factors, such as anxiety and fear of pain, and non-psychological factors, such as modifying throwing mechanics to avoid pain. Therefore, we could not classify these players into either the psychologically or non-psychologically triggered yips groups, and they were handled as an independent group. Although this group comprises a small proportion of yips-affected athletes, repeated painful throwing can trigger yips. Therefore, it is important to avoid throwing in painful conditions and to treat these conditions as soon as possible to prevent the development of yips.

Among the 25 players in the non-psychological triggered yips group, 3 had triggers to throwing yips owing to changes in throwing mechanics. This result indicated that a change in throwing mechanics is a trigger for the initial onset of yips. This finding is consistent with the features reported in patients with focal dystonia. Sadnicka et al. (2016) reported that in patients with musician's dystonia, a slight technical change may trigger the onset of symptoms. In addition, previous studies investigated the relationship between yips and “Reinvestment,” which is defined as consciously attempting to control movements by utilizing explicit and rule-based knowledge during skill execution (Masters and Maxwell, 2008). Watanabe et al. (2021) speculate that reinvestment may be involved in yips, based on the fact that alpha-band event-related desynchronization during precision force control task in athletes with yips is different from control individuals. Therefore, results of the present study, in which throwing yips occurred in some players who were in the process of consciously changing their throwing mechanics, would be reasonable in light of previous studies.

We were unable to identify the trigger of the initial onset of yips in the majority of players belonging to the non-psychological triggered yips group. Considering the triggers proposed in focal dystonia (Altenmüller and Jabusch, 2010), it is possible that non-psychological factors such as genetic predisposition and changes in sensory input, which were not investigated in this study, might also be involved in the initial onset of yips.

The results of the personality traits indicate that the agreeableness score was significantly higher in the psychologically triggered yips group compared with the non-yips group. Conversely, there was no significant difference in agreeableness scores between the non-psychologically triggered yips and the non-yips groups. These results suggest that the relationship between agreeableness score and yips differed depending on the trigger of yips. Few reports have evaluated the relationship between yips and agreeableness. Recently, Clarke et al. (2015) investigated the relationship between yips and personality traits examined by the BFI-10, and reported that there was no significant difference in the agreeableness score between the presence and absence of yips. We believe there are two major reasons for this discrepancy between the previous and present studies. The first is the differences in the sports investigated in each study. Clarke et al. (2019) studied the yips in golfers and archers, which are individual sports, whereas we studied the throwing yips in baseball, which is a team sport. Prior works have shown that agreeableness, which is one of the big five personality traits, differs between team sport and individual sport athletes (Nia and Besharat, 2010). Furthermore, unlike individual sports such as golf or archery, throwing errors in baseball affect not only oneself but also the throwing partner and the victory or defeat of the team. Of the 19 players classified into the psychologically triggered yips group in this study, most (16 players) were aware that the symptoms of yips were initially triggered by throwing errors or anxiety about throwing. Moreover, previous studies reported a positive correlation between agreeableness scores and sense of guilt (Einstein and Lanning, 1998; Abe, 2004). These findings suggest that the higher the agreeableness score, the more likely the player is to feel guilty when the team loses or for causing trouble for other players due to their throwing errors. Therefore, we speculate that players with higher agreeableness scores are more likely to experience psychological conflicts due to the guilt and anxiety associated with throwing errors, which may lead to psychologically triggered yips.

The second is the difference in how yips were classified in each study. This study used a trigger-based classification of yips, whereas Clarke et al. (2015) classified golfers and archers into yips and choking groups based on their symptoms and showed no significant differences in agreeableness scores with or without each symptom. Therefore, the difference in the classification of yips may have led to the discrepancy between these results.

The agreeableness score of the psychologically triggered yips group was significantly higher than that of the non-yips group, but not significantly different from that of the non-psychologically triggered yips group. Therefore, it is possible that the non-psychologically triggered yips group also included some players with high agreeableness scores. Since the reason for this cannot be mentioned from the results of this study, further investigation with a larger sample size will be necessary.

The neuroticism score was significantly higher in the non-psychologically triggered yips group than in the psychologically triggered yips group. This result suggests that the neuroticism score clearly differs depending on the trigger for the initial onset of yips. Studies on the association between yips (or focal dystonia) and neuroticism have reported conflicting results. Enders et al. (2011) reported that the neuroticism score was significantly higher in patients with musician's dystonia than in healthy musicians or non-musicians. The perfectionism and anxiety scores, which are closely related to neuroticism, are also high in both yips and choking (Stinear et al., 2006; Roberts et al., 2013). However, Clarke et al. (2019) reported that there was no significant difference in neuroticism scores between yips (and choking)-affected athletes and non-affected athletes. Therefore, the results of the present study, in which the neuroticism score varies according to the triggers of the onset of yips, were not inconsistent with these previous studies. Future work is necessary to investigate the relationship between classification based on the triggers of yips and indicators, including anxiety and perfectionism, which are closely related to neuroticism.

The significantly higher agreeableness score in the psychologically triggered yips group and the higher neuroticism score in the non-psychologically triggered yips group suggest that there may be different triggers for the initial onset of the yips depending on players' personality traits. As mentioned above, yips are classified into Type I and Type II according to their subjective symptoms, which contributes to their treatment selection. However, classification based on the trigger of yips has not been reported thus far. The results of this study, which show for the first time an association between the trigger-based classification of yips and personality traits, have important implications from a preventive perspective because of their potential relevance to the mechanism of development of yips. However, it remains controversial whether these personality traits in yips-affected players are preexistent or a psychoreactive phenomenon (Jabusch and Altenmuller, 2004; Enders et al., 2011). Since all studies that have investigated the relationships between yips (or focal dystonia) and personality traits, including the present study, are cross-sectional studies (Hughes and Mclellan, 1985; Enders et al., 2011; Amouzandeh et al., 2017; Clarke et al., 2019), no clear conclusions can be drawn on this argument. We support the view that the personality traits in yips players are preexistent phenomena rather than psychoreactive, as reported by Altenmüller and Jabusch (2009) and colleagues. This is because if the personality traits observed in players with yips are psychoreactive phenomena, similar personality traits should be obtained regardless of the trigger of the initial onset, whereas the personality traits of the two groups categorized based on the trigger-based classification used in the current study differed. In the future, large prospective studies are necessary to clarify the causality between personality traits and the initial onset of yips.

When assessing the relationship between yips classification and symptom progression, the non-psychologically triggered yips group tended to have a more gradual onset compared with the psychologically triggered yips group. This difference may exist because the psychologically triggered yips group is often triggered by a specific episode such as a throwing error and anxiety about throwing or reprimand from others, while the non-psychologically triggered yips group is often triggered by the absence of a clear episode. These findings support the possibility that psychologically triggered yips and non-psychologically triggered yips are caused by different mechanisms.

Using GLMM, we investigated whether trigger-based classification of yips and throwing distance were associated with symptom severity, i.e., frequency of throwing errors at the time when symptoms of yips were most severe. We found that the frequency of throwing errors was significantly higher for short-distance throwing (< between base distance) than for long-distance throwing (> 50 m). In yips-affected golfers, a previous study reported that the symptoms of yips are more likely to occur in short putts of ~1–4 feet (McDaniel et al., 1989). These findings suggest that the distance to the target is one of the crucial factors affecting the severity of the symptoms of yips. Although the reason for the paradoxical symptoms of yips, in which symptoms are stronger when the distance to the target is short, is unknown, we speculate that excessive muscle contraction or co-contraction observed in yips-affected athletes (Stinear et al., 2006; Adler et al., 2011, 2018) may contribute to the failure of short-distance throwing or putting, which requires control by relatively weak muscle activity. The frequency of throwing errors was significantly higher in the non-psychologically triggered yips group than in the psychologically triggered yips group. These results suggest that the severity of the symptoms of the yips depends on the trigger for the initial onset of the yips. Because there is often no clear trigger for the initial onset of non-psychologically triggered yips, it is difficult to discuss the prevention of yips based on the present results. However, since excessive practice is thought to have an adverse effect on the onset and progression of task-specific dystonia (Byl et al., 1996; Byl, 2007), it is important to detect symptoms and rest during the early stage of symptoms to prevent symptom progression. Thus, the present finding that the typical symptoms of yips are associated with difficulties in short-distance throwing may be helpful in determining the presence or absence of yips and in considering ways to prevent symptom progression.

Prior work on the relationship between psychological situations and yips symptoms indicated that yips affected golfers were more likely to show yips symptoms under conditions of high psychological stress, such as tournaments (McDaniel et al., 1989; Smith et al., 2000). Therefore, we predicted that the psychologically triggered yips group was more likely to show symptoms, particularly in more stressful psychological situations such as games. However, there were no significant differences between the psychologically and non-psychologically triggered yips groups regarding whether symptoms worsened during games compared with practice. This result suggests that even in yips-affected baseball players triggered by non-psychological factors, psychological pressure such as games can aggravate symptoms. Smith et al. (2003) reported psychological anxiety to be a factor in exacerbating yips symptoms, regardless of the type of yips (choking or dystonia). Therefore, to improve yips symptoms, treatment such as cognitive behavioral therapy for relieving anxiety related to the nervous situation may be required regardless of the triggers or symptoms of yips.

One of the limitations of this study is that we used a trigger-based classification of yips, but it is not clear whether this classification is related to the symptom-based classification (Types I and II yips) (Smith et al., 2003). Symptom-based classification of yips has not been established in baseball. Previous studies in golfers have shown that the classification of yips by subjective symptoms is validated by physiological indicators using electromyography (Stinear et al., 2006; Adler et al., 2018). Therefore, we must establish a symptom-based classification of yips in baseball using both subjective and physiological indices to investigate its relationship with the trigger-based classification of yips. Second, the present study did not obtain standardized measures related to anxiety, fear, or perfectionism. We need to conduct cluster analysis by adding these measures to the NEO-FFI to establish a detailed classification of personality traits. Furthermore, longitudinal studies are required to clarify the causal relationship between classification and initial onset of yips symptoms.

In conclusion, we investigated whether the symptoms of yips and personality traits differed according to the trigger-based classification of yips. We obtained novel findings indicating that the psychologically triggered yips group showed significantly higher agreeableness scores compared with the non-yips group. Additionally, the non-psychologically triggered yips group showed significantly higher neuroticism scores and a higher frequency of throwing errors, particularly in long-distance throwing, compared with the psychologically triggered yips group. Since trigger-based classification of yips is closely related to yips symptom strength and the personality traits of players, the results of this study may contribute to a better understanding of symptoms of throwing yips and the establishment of prevention for yips. In the future, large prospective studies are necessary to investigate the causal relationship between personality traits and the trigger for the initial onset of yips.

## Data Availability Statement

The raw data supporting the conclusions of this article will be made available by the authors, without undue reservation.

## Ethics Statement

The studies involving human participants were reviewed and approved by Local ethics committee of the Ibaraki Prefectural University of Health Sciences. The patients/participants provided their written informed consent to participate in this study.

## Author Contributions

TA, KA, HS, and TK designed the study. TA, KK, and TK collected the data. All authors contributed to data analysis, interpretation, and write the manuscript. All authors contributed to the article and approved the submitted version.

## Conflict of Interest

The authors declare that the research was conducted in the absence of any commercial or financial relationships that could be construed as a potential conflict of interest.

## Publisher's Note

All claims expressed in this article are solely those of the authors and do not necessarily represent those of their affiliated organizations, or those of the publisher, the editors and the reviewers. Any product that may be evaluated in this article, or claim that may be made by its manufacturer, is not guaranteed or endorsed by the publisher.
